# Prediction of the ORC Working Fluid’s Temperature-Entropy Saturation Boundary Using Redlich-Kwong Equation of State

**DOI:** 10.3390/e20020093

**Published:** 2018-01-30

**Authors:** Axel Groniewsky, Attila R. Imre

**Affiliations:** 1 Department of Energy Engineering, Budapest University of Technology and Economics, Muegyetem rkp. 3, H-1111 Budapest, Hungary; 2 MTA Centre for Energy Research, Department of Thermohydraulics, P.O. BOX 49, H-1525 Budapest, Hungary

**Keywords:** Organic Rankine Cycle, isochoric heat capacity, vibrational degree of freedom

## Abstract

The shape of the working fluid’s temperature-entropy saturation boundary has a strong influence, not only on the process parameters and efficiency of the Organic Rankine Cycle, but also on the design (the layout) of the equipment. In this paper, working fluids are modelled by the Redlich-Kwong equation of state. It is demonstrated that a limiting isochoric heat capacity might exist between dry and wet fluids. With the Redlich-Kwong equation of state, this limit can be predicted with good accuracy for several fluids, including alkanes.

## 1. Introduction

When a heat source is limited in temperature or in thermal power, it becomes attractive to adopt an Organic Rankine Cycle (ORC) [1,2] to explore it. This technology uses organic fluids instead of water, which makes it suitable for power generation based on any source, including solar, geothermal, biomass or waste heat. Ever since this technology was introduced, the choice of a suitable working fluid plays a key role in the application. Optimization algorithms not only involve design parameters as in conventional Rankine Cycles [3], but working fluids as well [4,5]. Working fluid selection criteria generally include thermodynamic and physical properties, chemical stability in the desired temperature range, compatibility, environmental impacts, safety, and production costs, which also refers to the availability of the fluid. Among the thermodynamic properties, the shape of the saturated vapour curve on the temperature-entropy plane has a significant impact on the ORC system, modifying the operational variables as well as altering the configuration and design of the equipment.

**Figure 1:**
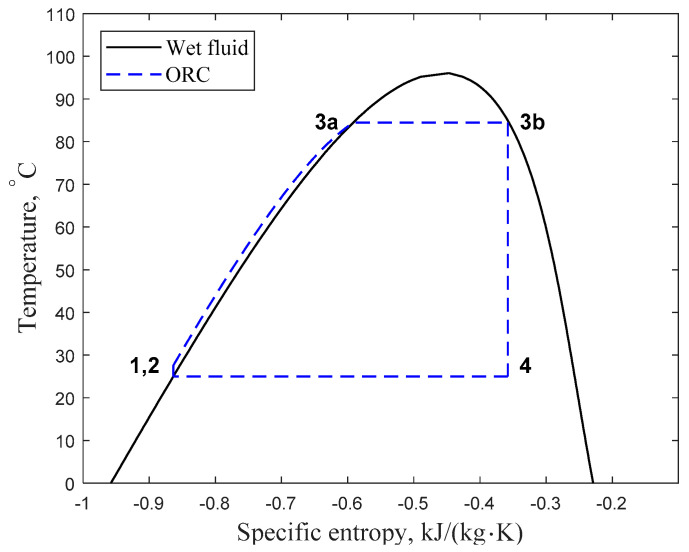
Schematic *T-s* diagram for a wet working fluid: 1,2 adiabatic compression of low pressure liquid to high pressure liquid; 2 → 3a → 3b isobaric heating of subcooled liquid (2 → 3a) and complete evaporation to saturated vapour (3a → 3b); 3b → 4 adiabatic expansion of high pressure vapour to low pressure “wet vapour”; 4 → 1 isobaric cooling, changing the “wet vapour” to saturated liquid.

Based on the slope (d*s*/d*T*) of the saturated vapour line on *T-s* (temperature vs. specific entropy) diagram, working fluids are characterized as wet, isentropic or dry with negative, zero or positive gradients, respectively [6]. From a theoretical point of view, however, the isentropic and dry categories can be merged [7]. When saturated vapour expands in an adiabatic and reversible manner, a wet working fluid ends up in the two phase region (coexistence region). In cases like this, vapour leaving the turbine contains a significant amount of droplets. Therefore, systems with this type of working fluids require a superheater to prevent turbine blade erosion, which makes them less appealing for low grade waste heat recovery. However, isentropic and especially dry types of working fluids mostly end up in the superheated region, which can increases the burden of the condensation process. A schematic *T-s* diagram for a wet working fluid can be seen in Figure 1; where various states are numbered to show the processes of a simple Rankine cycle. In this case, the adiabatic expansion (3b → 4) ends in the “wet vapour” region, containing vapour mixed with droplets.

The amount of literature in which the authors identify the most appropriate fluid for a given temperature range is significant, yet only a handful of papers present general solutions for any kind of heat source or deal specifically with the geometry of the working fluid’s *T-s* diagram.

Using Trouton’s empirical rule, Garay [8] concluded that fluids having two or more atoms in their structures and a molecular weight of more than 160 kg/kmol have positive slopes. A theoretical study by Goldstein [9] however showed that the slope of the saturated vapour line is—to a first approximation—a function only of the number of atoms in the molecules and not of their weight or character. Therefore, molecules with small numbers of atoms would be wet, whereas, for molecules with large numbers of atoms, they would be dry. Isentropic fluids require the number of atoms to be between five and ten [9,10,11]. Morrison [12], when he was investigating the shape of the phase boundary of working materials of refrigerators and heat pumps came to the conclusion that the differences in geometry of the *T-s* diagram over low to moderate pressure ranges is a consequence of molecular structure. Liu et al. [13] derived an expression to compute the slope of the saturation vapour curve and came to the conclusion that hydrogen bond in certain molecules, such as water, ammonia, and ethanol results in wet fluids due to larger vaporizing enthalpy. Chen et al. [6] however pointed out that the formula was developed through simplifications and was verified at the fluids’ normal boiling points, resulting some large deviations at off-normal boiling points. In their paper they also analyzed and discussed the thermo-physical properties of working fluids, deriving generalized conclusions. Quoilin et al. [14] gave guidelines and indicators for selecting the most appropriate working fluid, concluding that the thermodynamic efficiency alone cannot be considered as the sole criterion for the selection, instead a multi-objective selection method is required. Su et al. [15] employed an artificial neural network to establish the relationship between the molecular structures and the saturated vapour slope of working fluids. The nature of the saturated vapour line was represented by the slope angle (inverse tangent of vapour slope) and correlated with the reduced temperature, the molecular weight, 10 molecular groups—which cover most working fluids employed in thermodynamic cycles—and a topological index. Invernizzi et al. [16] defined molecular complexity and showed that the higher the molecular complexity is, the dryer the working fluid becomes. Garrido et al. [17] created a dimensionless function, reducing the customary classification to a criterion based on how the new functions value compares to the value of the isobaric heat capacity of the ideal gas. Applications were presented for cubic models, specific multi-parameter equations, molecular based models, and virial density expansions. From their results they also concluded that dry behavior depends on the number of atoms that compose the molecule, and it is generally observed in long-chained molecules.

Although most of these studies come to the correct conclusion within the range of their examination, it is difficult to benefit from these results by practicing engineers. Groniewsky et al. [18] however introduced a simple thumb-rule, based on the internal degree of freedom—related to the number of atoms in the molecule—and on the isochoric heat capacity of saturated vapour to predict the type of the working fluid. By using van der Waals (vdW) equation of state they concluded, that molecules with higher active degrees of freedom are probably dry or isentropic. The applicability of the rule was demonstrated on linear alkanes, yet the accuracy of heat capacity limit for wet-to-dry transition was limited, as vdW provides only qualitatively rather than quantitatively correct solution. The aim of this paper is to give better prediction for wet-to-dry transition, using Redlich Kwong (RK) equation [19] on a reduced T-s plane.

Obviously, there are several equations of state (EOS) in use and we do not have the intention to apply this method for all of them, but we do believe that the comparison of the single VdW with a mathematically only slightly different, yet physically more accurate cubic EOS is necessary at least for two reasons. First, in this way we can test the sensitivity of our method by applying different EOS. Second, by using EOS which can predict vapour phase properties for a certain group of materials (like RK for lower linear alkanes) we could test not only the qualitative but also the quantitative accuracy of this method.

## 2. Methods

For demonstrating the sensitivity of the method to the applied EOS we were looking for an EOS with higher accuracy and similar mathematical formula than vdW. Since the equation of RK only differs from vdW in the denominator of its second term, yet provides a more precise solution for lower alkanes it became an ideal choice:

(1) $$p=\frac{R\cdot T}{v-b}-\frac{a}{\sqrt{T}\cdot v\cdot \left(v+b\right)}$$

where *p* is the pressure, *T* is the temperature, *R* is the specific gas constant, *v* is the specific volume, and finally *a* and *b* are material dependent parameters. A thermic EOS is necessary but not sufficient for calculating the *T-s* saturation boundary of pure substances, some caloric information is also required. This information is also contained in the specific heat capacity. Specific heat capacity, at constant volume can be expressed as:

(2) $$c_{v}(v,T)=c_{v}(v_{0},T)+T\cdot \int_{v^{\prime }=v_{0}}^{v}\frac{\partial^{2}p(v^{\prime },T)}{\partial T^{2}}\cdot dv^{\prime }$$

where *c_v_* is the isobaric heat capacity, while subscript 0 refers the initial or reference state. Since the second term of this expression is derived from the EOS, the required caloric information is embedded in the first one, which is a contribution of the ideal gas. To avoid any changes on the shape of the saturation boundary other than the one caused by RK, the ideal gas dependent part of the specific heat capacity had been chosen to be the same as previously for vdW-fluid [16], giving:

(3) $$c_{v}(v,T)=\frac{f}{2}\cdot R-\frac{3\cdot a}{4\cdot T^{3/2}\cdot b}\cdot \log\left(\frac{v\cdot \left(b+v_{0}\right)}{v_{0}\cdot \left(b+v\right)}\right)$$

where *f* is the internal degree of freedom; with the assumptions that *R*, *a* and *b* are positive coefficients. In this model, the constancy of *f* is assumed, which is not true for real systems, since vibrational degrees of freedom can be activated at higher temperatures. In this work however, which is meant to test only the sensitivity of the method for different EOS, we do not want to include any experimentally determined and fitted *f(T)* function, therefore we are keeping it temperature-independent.

Since *a* and *b* only influences the vertical and horizontal stretch of the phase boundaries—by alteration of the position of critical point—not the shape, we extended the solution by using reduced properties, with the resulting reduced pressure function:

(4) $$p_{r}=\frac{11.542\cdot T_{r}}{3.8473\cdot v_{r}-1}-\frac{14.8019}{\sqrt{T_{r}}\cdot v_{r}\cdot \left(3.8473\cdot v_{r}+1\right)}$$

where the subscript *r* refers to reduced quantities (divided by the corresponding critical quantity). To determine the phase boundaries of the coexistence region on the *p-v* diagram, we applied Maxwell construction and used *s = s*(*v,T*) function to get the slope of the saturation vapour curve on the *T–s* diagram:

(5) $$s(v,T)=s_{0}+{\int_{v^{\prime }=v_{0}}^{v}\frac{\partial p(v^{\prime },T^{\prime })}{\partial T^{\prime }}|}_{T^{\prime }=T}\cdot dv^{\prime }+{\int_{T^{\prime }=T_{0}}^{T}\frac{c_{v}(v^{\prime },T^{\prime })}{T^{\prime }}|}_{v^{\prime }=v_{0}}\cdot dT^{\prime }$$

marking the variable quantities with apostrophes.

One should be aware, that for more accurate description of a particular ORC working fluid more sophisticated EOS should be used (for example Backbone EOS [20]), but for general trends, simple ones like RK equation can be sufficient. Also, for multiphase systems, the calculation of *T-s* diagrams is more difficult, due to the different composition of the vapour and liquid phases [21].

## 3. Results

When the saturation boundary is calculated, the initial (lowest temperature) state of the working fluid corresponds to the triple point condition. Since every fluid has a different triple point, they have—even with the same caloric information (heat capacity function)—different reduced *T-s* diagrams. As the triple point does not appear in the RK equation directly and no particular fluid is calculated, the reduced temperature for the initial state has been chosen to be 0.45. Considering that several investigations in this area are lead in the reduced temperature range of 0.6–1 [15] our range appears to be a reasonable interval (for example for butane, this range corresponds to −18 °C to 152 °C, covering the range of most geothermal application). Besides, at lower temperature range working fluids tend to have lower density, which increases unit size and investment costs, making the practical significance of this temperature range low.

**Figure 2:**
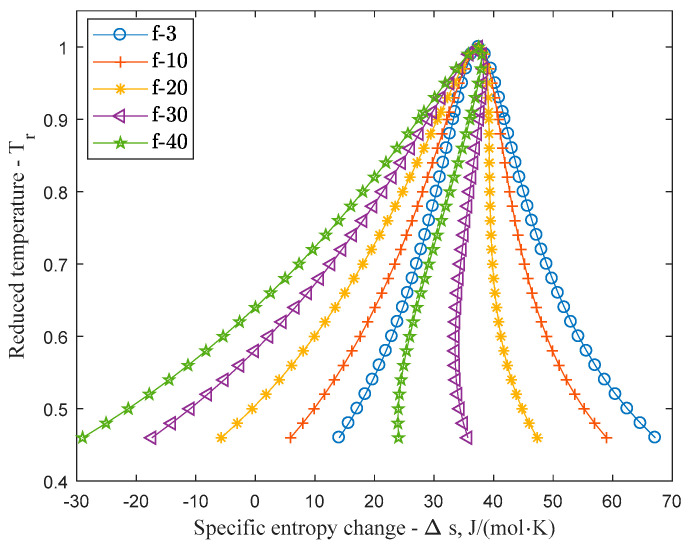
Reduced temperature-entropy saturation boundaries of Redlich-Kwong fluids with different internal degree of freedom, shifted to unified critical point.

As Equation (5) indicates, the entropy function depends on *s_0_* initial state, volume change and temperature change. Since the initial state does not influences the slope of the entropy function rather moving the saturation boundary horizontally, effecting all functions with different caloric information in the same manner, Figure 2 shows the phase diagrams calculated by using RK equation with different caloric state equations, shifted to unified reduced critical point for better comparison.

**Figure 3:**
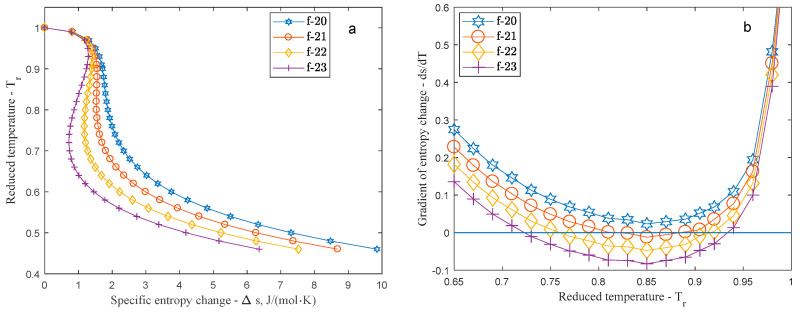
(**a**) Wet-isentropic-dry transition of Redlich-Kwong fluids (showing only the saturated vapour side of the *T-s* diagram) with internal degree of freedom between 20 and 23 on a reduced *T-s* plane; (**b**) The actual gradient of their entropy change between the reduced temperature range of 0.65 and 1. Zero value of the gradient represents isentropic behaviour.

Considering that the volume dependent entropy change (second term) of Equation (5) only influenced by the thermic equation, all change in the slope is the result of the caloric information. As the third term of Equation (5) indicates, the more dominant the caloric information compared to the thermic one, the dryer the character of the working fluid becomes. With increasing internal degree of freedom the shape of the phase changes and from wet (*f* < 20) it slowly becomes isentropic (19 < *f* < 23) and finally dry (*f* > 25). Figure 3a displays the transition between wet and dry slope and Figure 3b shows the gradient of the entropy change in the 0.65–1 reduced temperature interval. As Figure 3b shows the slope becomes isentropic (d*s*/d*T* = 0) first around 0.85 reduced temperature when *f* = 21.

**Figure 4:**
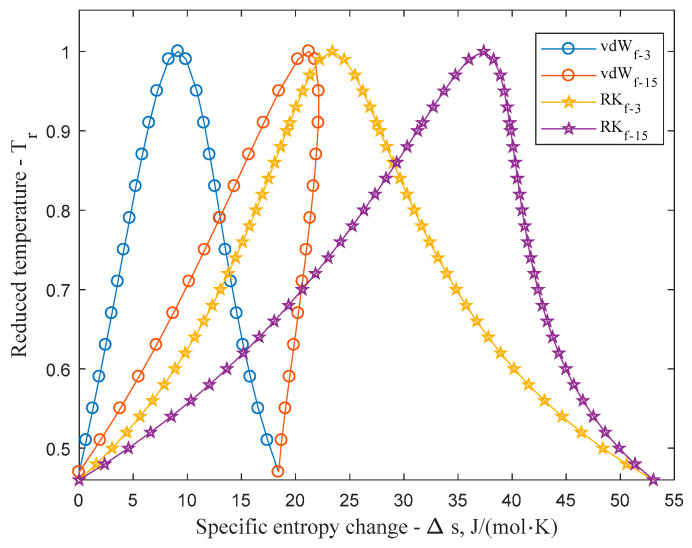
Reduced temperature-entropy saturation boundary of van der Waals and Redlich-Kwong fluids at lower (*f* = 3) and at higher (*f* = 15) degree of freedom. Comparison shows that RK fluids are less sensitive to the change of internal degree freedom than vdW fluids.

Figure 4 compares the reduced temperature-entropy saturation boundaries of vdW and RK equations at lower (*f* = 3) and at higher (*f* = 15) degree of freedom. Calculations at low degree of freedom show that the entropy change influenced by the thermic equation is much less significant for vdW than for the RK equation. Since the second term of Equation (5) is relatively small for the vdW equation it also means that the third, caloric information dependent term will dominate the outcome of the entropy function. Since RK equation has a higher influence on entropy change however, it reduces the contribution of the caloric information to the entropy function, causing the RK equation to remain wet even at higher (*f* = 15) degree of freedom. 

As the same volume change causes higher entropy change for RK than for vdW equation, it is less sensitive to the degree of freedom (caloric information) and the transition between wet and dry slope appears at a higher value. One can roughly say that a fluid is dry or isentropic, when its degree of freedom goes above *f* = 20...22 and therefore the isochoric heat capacity in the temperature range of interest goes above the range of (20/2)/*R*–(22/2)/*R*, i.e., 83.1–91.5 J/mol·K.

**Figure 5:**
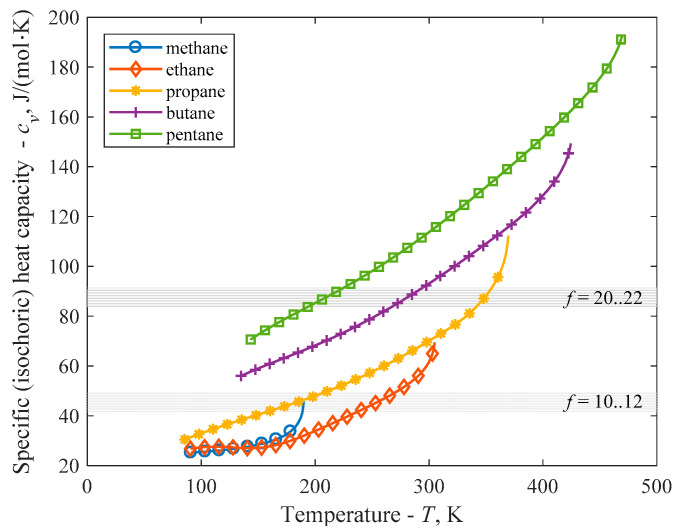
Isochoric heat capacities of lower alkanes (from methane to pentane) in their liquid range, compared to the dry-to-wet transition value based on vdW (*f* = 10–12, *c_v_* = 41.6–49.9 J/mol·K) and RK (*f* = 20–22, *c_v_* = 83.1–91.5 J/mol·K) equations.

For demonstration purposes, in Figure 5 one can see the real isochoric heat capacities of lower alkanes (from methane to pentane) in their liquid range (from triple point to critical point) [22] compared to the dry-to-wet approximate transition value established here by RK (*f* = 20...22, *c_v_* = 83.1–91.5 J/mol·K) and earlier by vdW (*f* = 10...12, *c_v_*=41.6–49.9 J/mol·K) [16] equations, marked by grey bands. For methane and ethane, *c_v_* cannot reach the limiting value, therefore it is expected to be wet. For propane, at high temperature the *c_v_* is over the limiting value suggesting transition to isentropic. Although propane is wet, but not too far from the isentropic, while butane and pentane are dry, this example shows that degree of freedom and vapour isochoric heat capacity might be used with success to separate wet and dry working fluids from each other, at least for systems, where RK equation of state can describe the vapour side of the saturation curve with proper accuracy (for example alkanes). Based on preliminary results, this method also can be used to distinguish between various sub-groups of dry and isentropic fluids [23].

It should be mentioned here that the model of Garrido et al. [17] came to the same conclusion placing the wet to dry transition between propane and butane, yet the application of the dimensionless *Ψ* function seems to be more challenging for practicing engineers than the use of *f* or *c_v_*. Since the estimation of degree of freedom at low-temperature is a simple task from the molecular structure and isochoric heat capacities are tabulated for various materials, these quantities seems to be more easily applicable to categorize fluids than the *Ψ* function.

## 4. Discussion

For working fluids used in Organic Rankine Cycles, the shape of the saturated vapour side of the temperature-entropy diagram is very important. There are several papers dealing with the problem by predicting the slope of this curve for various fluids, but most of them are not sufficiently accurate or too difficult to apply for engineering applications. In this paper, a simple model is presented, using the Redlich-Kwong equation of state; a cubic equation of state, which is still simple enough mathematically, but accurate enough to describe the vapour properties of small nonpolar compounds, like smaller alkanes. The wet-to-dry transition by going from smaller molecules to bigger ones (i.e., increasing the molecular degree of freedom) had been demonstrated; additionally a limiting degree of freedom (and a corresponding isochoric heat capacity value) were given as *f* = 21 and *c_v_* = 87.3 J/mol·K. For working fluids where heat capacity stays below this value in the entire two-phase range, wet characteristic can be expected, while for the rest, one might expect dry characteristic. It seems that the sensitivity of this procedure is quite high because by using only slightly different EOS the limiting *c_v_* and *f* were significantly different. Yet, these values were more accurate obtaining them from a more realistic EOS. Obviously, these numbers are only as good as the Redlich-Kwong equation, but they seem to be applicable for linear alkanes and for other simple fluids. This method can also be used for more sophisticated classifications than the traditional wet-isentropic-dry one [23]. Since the change of the geometry of *T-s* curves can be easily tracked by changing the molecular degree of freedom, characteristic extrema (local entropy minimum and maximum on the saturated vapour side) can be easily determined and various sub-categories within dry and isentropic classes can be revealed.
